# The accurate determination of Perfluorooctane sulfonic acid (PFOS) removal efficiency by integrated-sonochemical system

**DOI:** 10.1016/j.ultsonch.2025.107222

**Published:** 2025-01-03

**Authors:** Debabrata Panda, Maxime Cochennec, Stéfan Colombano, Benjamin Laulier, Pascal Tierce, Alexandre Baudouard, Sebastian Bristeau, Anne Togola, Julie Lions, Nicolas Devau, Eric D. van Hullebusch

**Affiliations:** a BRGM, 3 Avenue Claude-Guillemin, BP 36009, 45060 Orléans, France; b SinapTec, 7 avenue Pierre et Marie Curie, 59260 Lezennes, France; c Université Paris Cité, Institut de Physique du Globe de Paris, CNRS, F-75005 Paris, France

**Keywords:** Sonochemical, PFOS, PFAS, Frequency, E_EO_, Removal, Mineralization

## Abstract

Perfluorooctanesulfonic acid (PFOS) is one of the most investigated Per- and polyfluoroalkyl substances (PFAS) for being the strongest compound to eliminate and having adverse health concerns. In this work, we have conducted the sonochemical treatment of PFOS simulated water under high (500 kHz) and low (22 kHz) frequencies while monitoring the operational parameters via an integrated sonochemical system. The integrated advanced sonochemical system includes software to monitor treatment power, solution temperature and frequency while allowing distinctive control of the reaction conditions. Considering the lack of calorimetric measurements in earlier studies and the difficulty in achieving comparative outcomes, precise calorimetric measurements and determination of electrical energy per order (E_EO_) were performed in this study. The complete PFOS removal was achieved under 500 kHz frequency with optimum parameters including initial pollutant concentration (5 mg/L), ultrasound power density (400 W/L) and solution temperature (25 °C) within 180 min of treatment. The removal and mineralization extents (defluorination) were determined by ultra-high performance liquid chromatography–mass spectrometry (UPLC-MS/MS) and ion-chromatography (IC) analysis. Under optimum conditions, 100 % removal and 99 % mineralization were achieved. The rate constant (k) ranged from 0.011 to 0.031 ${min}^{-1}$ (first-order reaction), which increased with the increase in the power density. While the solution temperature did not significantly affect the PFOS removal efficiency, the initial concentration was found to have a prominent effect on the reaction rate constant. However, experiments at low frequency (22 kHz) showed negligible removal efficiency. The specific energy requirement for reaching 90 % removal while considering the power consumed by the ultrasonic system from the main electrical source was determined to be 700 $kWh/m^{3}$, which is much lower than other reported work under similar conditions. This work will be useful for both laboratory and industrial upscaling while acting as a benchmark reference to follow.

## Highlights

• The overall PFOS elimination efficiency has been accurately determined.
• The integrated ultrasound system is found to be highly efficient compared to earlier reports.
• Complete removal of PFOS is possible under optimum reaction conditions.
• PFOS mineralization is driven by thermal decomposition at the bubble interfacial region.

## 1 Introduction

Perfluorooctanesulfonic acid (PFOS) is one of the extensively used per- and polyfluoroalkyl substances (PFAS) in consumer products and industrial applications, due to its unique chemical and physical properties [1]. PFOS has been found in a wide range of industrial applications related to hydraulic fluids, textiles, hard and decorative chromium plating, medical imaging and fire-fighting foams (AFFFs), which has been still extensively used across the world following its ability to withstand extremely high temperatures, shield surfaces from moisture, oil, or abrasion and resistant to stains and fire. PFOS is linked to serious health and environmental concerns while not only limited to endocrine disruption, birth defects and cancer [2]. The PFOS was reported to be transported to the foetus through cord blood and to the infant through breast milk, which after a certain exposure limit could result in developmental effects in infants [3]. Adding to this, following the survey and news published by Le Monde in 2023 [4], [5], the presence of PFAS was identified in 23,000 contaminated sites across Europe including 2,300 sites at levels dangerous for health. The worldwide groundwater concentration of PFOS was reported to be in the range from 0.01 ng/L to 5 mg/L, which is highly concerning [6], [7]. However, it is nearly impossible to trace back the source of PFOS contamination in the environment due to its diverse application.

PFOS is one of the most widely studied PFAS for its removal. However, due to its distinctive physiochemical characteristics leading to a thermally stable structure, it’s challenging to be removed via conventional advanced oxidation-based wastewater treatment methods. Even though advanced oxidation-based methods such as ozonation [8], electrochemical treatment [9], photochemical [10] and their combined approach [11], have been used for its elimination, they are not cost-effective and efficient compared to cavitation-based treatment. Ozonation involves reagent costs with each application and may also require pH adjustment (alkaline ozonation) to enhance PFOS removal [8]. The electrochemical oxidation coupled with UV irradiation (E + UV) [10] demonstrated a high energy demand (300 W UV irradiation plus electrode electrical consumption) to achieve 99.6 % PFOS removal (initial concentration: 1 mg/L) within 120 min, making it unfeasible for upscaling. Recently, Marsh et al. [12] utilized a dual high-frequency (700 + 950 kHz) ultrasound system to treat PFAS having PFOS in the mixture. However, no calorimetry measurement was performed and the reason behind using two high frequencies together was not justified. Rodriguez-Freire et al. [13] treated PFOS (10–460 μM) via mega-sonic ultrasound (500 kHz–1 MHz). However, their reported power density (ca. 8 W/cm^2^) does not represent the actual value for comparison. Wood et al. [14] utilized a 500 kHz reactor to treat PFOS (10 mg/L) and achieved 93.8 % degradation within 4 hr of treatment. Nevertheless, there was no information on the power density and the mineralization extent. Shende et al. [15] implemented a 575 kHz ultrasonic reactor to degrade low PFOS concentration (100 nM) within 2 hr of treatment. Even though they mentioned the power density, the calorimetric yield was not provided. They also explored the inclusion of oxidants (iodate and chlorate) in an Argon environment. However, these oxidants proved ineffective in improving PFOS degradation under such conditions. Kewalramani et al. [16] employed high-frequency (700 kHz) transducers for the treatment of PFOS (11.4 uM). Vecitis et al. [17] implemented a sonication frequency of 354 kHz for low-concentration PFOS (200 nM) removal and even sparged all reactions with Argon for at least 30 min prior to the reaction. However, they did not include calorimetric yield or efficiency information. Moriwaki et al. [18] reported the sonochemical degradation of PFOS (10 mg/L) at an ultrasound frequency of 200 kHz while using both air and argon atmosphere. Under argon and air atmosphere, they reported to obtain a rate constant (k) of 0.016 ${min}^{-1}$ and 0.0068 ${min}^{-1}$, respectively. Cheng et al. [19] utilized 612 kHz sonication frequency to treat spiked groundwater samples consisting of PFOS (100 μg/L) and PFOA (100 μg/L) while sparging with argon 30 min prior to and throughout the reaction. They also compared the PFOS groundwater removal outcome to simulated (Milli-Q water) PFOS removal efficiency, which stands at a rate constant (k) of 0.0135 ${min}^{-1}$ vs 0.0192 ${min}^{-1}$, which is 70.3 % of the Milli-Q rate constant. Although researchers have investigated the use of an Argon atmosphere, it is not practical for industrial-scale PFAS treatment due to its high cost.

Overall, considering previous sonochemical treatments, it can be inferred that most of the studies apart from Ilić et al. [20], provided proper calorimetric measurement/yield and the electrical energy per order (E_EO_) value for realistic comparison. While certain studies used the G_Value_ method (G_Value_ = $\frac{V.(C_{0}-C_{t})}{P.t}$ g KWh^−1^), to determine the ultrasonic energy efficiency, the lack of accurate calorimetric power (P) information makes them prone to inaccurate determination. Therefore, even though high-frequency (200 kHz–1 MHz) ultrasound technology has shown to be effective in removing PFOS from the water matrix, the absence of proper calorimetric measurement and E_EO_ determination has hindered the understanding of the comparative effectiveness and energy efficiency. In addition, there is no thorough study on the PFOS mineralization process rather than just degradation, which is vital to optimize the reactor performance. As a result, the sonochemical technologies’ upscaling potential has become limited. Therefore, precise calorimetric measurement along with E_EO_ needs to be always provided, which could portray the overall performance of the sonochemical method and its viable application with respect to energy efficiency. In addition, only limited studies have reported the technology’s effectiveness in mineralizing the PFOS compound (i.e., including the production of F^-^), which is essential to understand if the pollutant is leaving behind any by-products in the treated matrix. It is also vital to accurately monitor the operating parameters to determine the optimum value for efficient removal, which is only possible via a real-time integrated system that eliminates measurement inaccuracies during experiments. For the first time, an integrated ultrasonic system with real-time operational parameters monitoring capability has been used in this work. Unlike other studies, this study reports calorimetric yield/efficiency and E_EO_, PFOS mineralization efficiency and the impact of other essential parameters to understand the upscaling potential.

The propagation of intense sound waves in liquids with a power ultrasound device can produce acoustic cavitation that involves the initiation of bubble growth, followed by intense collapse events. During the collapse, the enclosed energy in the cavitation bubble gets released into the surroundings while generating both sonophysical effects (microjets, microstreaming, shockwaves) and sonochemical effects (pyrolysis and radical generation) [21]. High-frequency (500–1000 kHz) power ultrasound is believed to mineralize PFOS via high-temperature pyrolysis at the bubble core and at the bubble–liquid interfacial region owing to its surfactant property. The collapse event could generate temperatures up to 5000  K, which is ideal for the pyrolysis of inert compounds such as PFOS.

The role of operational parameters such as initial pollutant concentration, ultrasound power density, solution pH and temperature have been evaluated in the current work. Also, the effectiveness of the low-frequency (22 kHz) treatment was compared with the high-frequency (500 kHz) treatment. The PFOS removal extent was determined by ultra-high performance liquid chromatography-tandem mass spectrometry (UPLC-MS/MS) analyses. The presence of degradation by-products was also proposed based on UPLC full scan mass spectrometry analysis. The mineralization efficiency was determined by ion-chromatography (IC) analysis by measuring fluoride evolution over the treatment time. Control experiments were also conducted to understand the PFOS adsorption extent onto the ultrasonic reactor wall.

## 2 Experimental

### 2.1 Materials

PFOS as perfluorooctanesulfonate potassium salt (C_8_F_17_SO_3_K, PFOS, 99 %), ammonium acetate (HPLC grade) and methanol (HPLC grade) were purchased from Fisher Scientific (France). The internal standard [Sodium Perfluoro-1-Octanesulfonate-^13^C_4_ (PFOS-^13^C_4_)] was provided by Wellington Laboratories Inc. (Guelph, Ontario, Canada). Agilent nylon syringe filter of 0.45 μm pore size and 5 mL syringes were purchased from Fisher Scientific (France). The fluoride standard (99.99 %) was purchased from Fisher Scientific (UK). Each chemical standard has a purity of >97 %. All aqueous solutions were prepared with high-purity water (Milli-Q water).

### 2.2 Sonochemical experiments

**Fig. 1:**
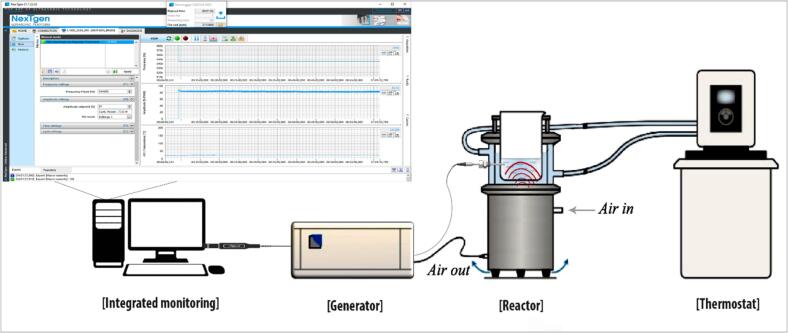
Schematic of the integrated ultrasonication system.

PFOS stock solution (200 mg/L) was prepared in Milli-Q water and was stored at 2 °C for subsequent experiment needs. For sonochemical experiments, the desired working solutions of concentration range from 2 µg/L to 5 mg/L were prepared following the dilution of the stock solution in Milli-Q water. Two different bath-type reactors (500 mL) having 22 kHz and 500 kHz frequency generators were used for conducting the experiments while being operated consistently with 300 mL reaction solution capacity. The jacketed reactors are made up of stainless steel (316L) while having a height of 114.4 mm and an internal diameter of 81 mm. The maximum external diameter is 119 mm. The total cooling volume within the reactor’s jacket is 197 mL. Control experiment without sonication was also conducted to determine if there is any loss of PFOS to the reactor surface. The reactor and ultrasound generator were connected to the NexTgen PC software, which is an innovative diagnosis tool for the ultrasound system. The complete set-up was custom-made by SinapTec, France. The NexTgen software makes it possible to select the operating mode of the generator (frequency and amplitude control) and to optimize all other operating parameters (reaction solution temperature, precise stop conditions) for steering the ultrasound process. The interface allows adjusting the generators’ parameters in perfect control and recording the operation data for later access of those results and traceability of the ultrasound system. The schematic of the ultrasonication experimental setup and the overview of the integrated software is presented in Fig. 1. Sonochemical treatments were conducted for up to 180 min and samples were collected at regular intervals for UPLC-MS/MS and IC analysis. The reaction solution temperature was maintained (25–35 °C) by a chiller having a water bath and recirculating capacity. The ultrasonic power density (200–400 W/L) and PFOS initial concentration (2 µg/L–5 mg/L) were varied to understand the optimal treatment conditions. The reaction pH was monitored continuously to understand the PFOS mineralization progress. To replicate the industrial application feasibility, all experiments were conducted under the air atmosphere rather than the argon atmosphere. Calorimetric measurements were performed to evaluate the power efficiency. To validate outcomes, individual experiments were repeated thrice.

### 2.3 Removal efficiency

The PFOS removal efficiency was calculated in terms of both UPLC-MS/MS and IC analysis outcomes. The mass spectrometry outcome was considered to calculate the removal efficiency with treatment time. After deciding the optimal operational parameters for PFOS removal, corresponding plots are constructed based on Eq. (1). The removal trend followed first-order reaction kinetics.

(1) $$\ln\left(C_{t}/C_{0}\right)=-k_{\mathrm{PFOS}}t$$

Where, k_PFOS_ (min^−1^), C_0_ and C_t_ represent rate constant and measured PFOS concentration at time “0″ and “t”, respectively.

To measure the PFOS mineralization efficiency, fluoride ion concentration was determined by ion chromatography. Percentage fluoride ion (F^-^) release was determined by Eq. 2, which demonstrated the reaction completeness, i.e., the percentage mineralization.

(2) $$\%\;F^{-}\;\;release=\frac{F^{-}}{{\mathrm{TOF}}_{0}}\;\times 100\%$$

Where,

[F^-^] = Fluoride ion concentration (mg/L).

[TOF_0_] = Total organic fluorine at time t = 0 (mg/L).

For example: PFOS contains 64.6 % fluorine by mass. Therefore, inserting [TOF_0_] as 64.6, which is present in PFOS at time zero, the percentage mineralization was found to be as high as 98 % for 5 mg/L PFOS under 500 kHz and 400 W/L power density operation.

### 2.4 Calorimetric measurements and determination of electrical energy per order (E_EO_)

The actual power delivered by the ultrasound system should be determined by calorimetric measurements, which rely on calculating the rate of reaction medium heat generation initiated by ultrasound waves irradiated into the system. To calculate the amount of thermal energy that the ultrasonic reactor added to the reaction solution relative to the energy that was drawn from the power grid, calorimetric measurements were carried out. The calorimetric measurements consisted of monitoring the solution temperature ($T_{s}$) every minute. The whole measurement did not last longer than 10 min in order to avoid heat losses as much as possible. The cooling was set off for the same reasons. The calorimetric power − $P_{c}$ [W] − can then be calculated based on Eq. (3).

(3) $$P_{c}=mC_{p}\frac{dT_{s}}{\mathrm{dt}}$$

Where, m [kg] is the mass of water, $C_{p}$ [J/kg K] is the specific heat capacity of water and $dT_{s}/dt$ [K/s] is the rate of solution temperature increase. With the known calorimetric power, the calorimetric yield (denoted r) is obtained as: $r=P_{c}/P_{\mathrm{el}}$, where $P_{\mathrm{el}}$ [W] is the electrical power. The electrical power was monitored using a wattmeter, which measured the instantaneous power delivered to the entire ultrasonic system (i.e., reactor and auxiliary components such as electronics and fans).

Electrical power is also used to determine the Electrical Energy per Order (E_EO_) parameter. The E_EO_ parameter [kWh/m^3^] expresses how much energy is required to achieve 90 % removal of a pollutant in one cubic meter of water. The Eq. (4) was used to calculate the E_EO_ parameter.

(4) $${\text{E}}_{\text{EO}}=\frac{P_{\mathrm{el}}}{V.k_{\mathrm{PFOS}}}$$

Where, $k_{\mathrm{PFOS}}$ [${min}^{-1}$] is the first order degradation rate constant and *V* [m^3^] is the solution volume.

### 2.5 Potassium iodide dosimetry

The cavitation activity can also be indicated by the potassium iodide (KI) dosimetry method, where the determination relies on the fact that iodine ions (I^−^) in an aqueous KI solution can be converted into iodine molecules (I_2_) under ultrasonic irradiation [22]. This iodine release serves as an indicator of acoustic cavitation. When the KI solution is irradiated, oxidation occurs, and I^−^ ions are oxidized by the generated hydroxyl radicals (OH•) to form I_2_. The excess I^−^ ions in the solution then react with I_2_ to produce triiodide ions (I_3_^−^). The concentration of I_3_^−^ ions can be measured using a UV spectrophotometer. Therefore, the dosimetry under varying frequencies was evaluated using KI dosimetry.

**Fig. 2:**
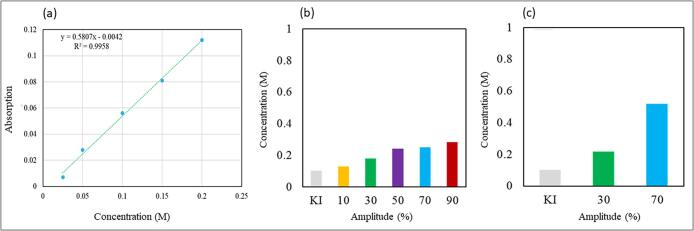
(a) Calibration curve of different concentrations of KI solution. Dosimetry measurements under (b) 22 kHz and (c) 500 kHz operation.

For this measurement, 15 mL of a 0.1 M potassium iodide solution was sonicated for 30 min at both low (22 kHz) and high (500 kHz) frequencies under varying amplitudes. After sonication, the absorbance of the solution was measured at 355 nm. A range of potassium iodide solutions with concentrations (0.025 to 0.2 M) were prepared and the absorbance of each concentration at 355 nm was measured to construct the calibration curve (Fig. 2). The number of hydroxyl radicals generated during the sonication process for iodide dosimetry can be calculated using the Beer-Lambert equation (Eq. 5) since the reaction between OH• and iodide is one-to-one (1:1) and the concentration of iodine (*c*) produced is directly proportional to the number of hydroxyl radicals generated.

(5) A=εlc

Where *A* is the absorbance (unitless), *ε* is the molar extinction coefficient (L/mol·cm), *l* is the path length in cm and *c* is the concentration in mol/L.

The dosimetry measurements (Fig. 2) showed that the concentration of I_3_^−^ ions increased under high frequency, while maintaining the same amplitude. Additionally, when using a specific sonicator, an increase in amplitude led to a corresponding rise in the concentration of I_3_^−^ ions. By inputting the molar extinction coefficient as 26,300 (L/mol·cm) under 355 nm, the path length of the solution in the spectrophotometer as 1 cm and the corresponding absorbance value from Fig. 2, it becomes evident that the amount of OH• radicals (c) generated under 500 kHz is twice the radicals generated under 22 kHz under the same electrical power.

### 2.6 Chemical analysis

#### 2.6.1 UPLC–MS/MS analysis

PFOS samples were diluted with a 50/50 water/methanol mixture to ensure that the PFAS levels in the samples were within the calibration range (5 to 5000 ng/L). A volume of 0.44 mL of the diluted sample was transferred to a polypropylene (PP) vial/cap. Additions of 10 µL of 5 % acetic acid diluted in water and 50 µL of an internal standard solution PFOS ^13^C_4_ at 2 µg/L were made prior to UPLC-MS/MS analysis. 10 μL were injected onto a WATERS Acquity UPLC for separation on a BEH C18 column (2.1 mm × 100 mm, 1.7 μm). A delay C18 column was used (isolator column 50 mm × 2.1 mm, Waters) to avoid PFAS contamination from the chromatographic system. Necessary precautions such as utilizing PFAS-free storage tubes and flushing the liquid chromatography system before analyses were followed to eliminate any false positives. The UPLC separation was carried out at a column temperature of 35 °C using a gradient composed of solvent A (5 mM aqueous ammonium acetate) and solution B (methanol 5 mM ammonium acetate). The gradient expressed as changes in solvent A was as follows: 0–6.5 min, 95 % to 5 % A; 6.5–7.0 min, 5 % A; 7.0–7.6 min, 5 % to 95 % A; 7.6–10 min, 95 % A. The flow rate was 300 μL/min. Considering the properties of PFOS (polarity, acidity, electronegativity), negative electrospray ionization (ESI) was chosen as the ion source interface and the LC sample was analyzed with a WATERS XEVO-TQXS (triple quadrupole). The electrospray conditions were as follows: Desolvation temperature 500 °C; Desolvation gas flow 1100L/h; Cone gas flow 150 L/h; Nebulizer gas flow 7 Bar; Capillary voltages −1000 V. Multiple reaction monitoring (MRM) mode was opted to quantify PFOS while considering the most abundant precursor/product ion transitions (PFOS: 499 → 80; PFOS ^13^C_4_: 503 → 80), whereas, for qualifiers, the second MRM transitions (PFOS: 499 → 99; PFOS ^13^C_4_: 503 → 99) were considered. The primary MRM transitions were used for the quantification, while the secondary transitions were used for the ion ratio confirmation to eliminate false positive results. The parameters of the MS segment were as follows: Cone voltage: 30 V and Collision energy: 37 eV. A good signal-to-noise ratio (S/N) was obtained after following essential precautions to avoid unwanted background contamination. Quantification was carried out by internal calibration using PFOS ^13^C_4_. PFAS quantification was based on 9-point calibration curves (5 to 5000 ng/L) having R^2^ > 0.99. In these conditions, LQ was estimated to be 20 ng/L (without considering sample dilution). Due to sample dilutions, the limit of quantification for PFOS in samples was 10 µg/L. For the detection of by-products, analysis in full scan mode (ESI+/- 50 to 600 *m*/*z*) was performed, using the same UPLC and mass spec parameters.

#### 2.6.2 IC analysis

The fluoride anion (F^-^) concentration was determined via suppressed conductivity ion chromatography with a Metrohm IC-940 system (Herisau, Switzerland). The chromatography system was fitted with a Metrohm Metrosep A Supp 7 analytical column (4 × 250 mm i.d.) and Metrosep C Trap1 guard column (4 × 30 mm i.d.). The IC separation was carried at 45 °C and the eluent consisted of 3.6 mmol/L sodium carbonate (Na_2_CO_3_). The flow rate was 0.8 mL/min and the run time was 10 min.

## 3 Results and discussion

### 3.1 Calorimetry and electrical energy per order (E_EO_)

**Fig. 3:**
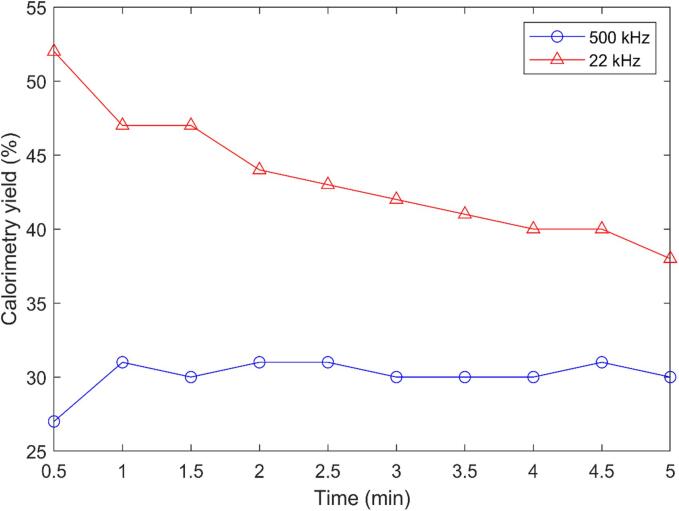
Calorimetric yield for 500 kHz and 22 kHz reactor.

The calorimetric yield for the 500 kHz ultrasonication test was found to be about 30 %, whereas the total electrical power was 80 W. It indicated that the acoustic power, i.e., the available power for the actual ultrasonication was 30.7 W. The calorimetric yield for both the frequencies (22 and 500 kHz) is presented in Table 1 and the yield efficiency for 500 kHz and 22 kHz are also shown in Fig. 3. This calorimetric yield was 3 to 6 times higher than the calorimetric yield measured by previous researchers under similar conditions [20]. The difference could be attributed to the unique ultrasonic generator used in our study, including the ability to precisely match the working frequency to the resonant frequency, thanks to the integrated software that allows real-time monitoring and modification of conditions.

**Table 1:** Calorimetric powers for 22 and 500 kHz frequencies at 80 W.

Frequency (kHz)	Applied frequency (kHz)	Applied power (AP) (W)	Calorimetric power (W)	Yield/efficiency (%)
22	22	80	42.2	43
500	544	80	30.7	30

Evaluating the ultrasound technology’s scale-up feasibility is dependent on its operational energy calculation. In 2001 [23], IUPAC recommended and approved the electrical energy per order (E_EO_) as the figure of merit for the comparisons among electrically driven advanced oxidation processes (AOPs). Ilić et al. [20] utilized a 580 kHz sonochemical reactor to treat GenX and PFOS. They reported obtaining a first-order degradation rate constant of k: 0.0153 min^−1^ for the 400 W/L power density with E_EO_ of 1644 kWh/m^3^. They also reported PFOS degradation under 200 and 300 W/L power density conditions. The E_EO_ was between 2340 (200 W/L) and 1644 (400 W/L) kWh/m^3^ for PFOS with an initial concentration of 1 mg/L. Considering our investigation, the E_EO_ for the 500 kHz–267 W/L ultrasonication test was determined to be 717 kWh/m^3^. We also conducted experiment under 500 kHz–400 W/L to compare the outcome and the reaction rate constant (k) was determined to be 0.031 min^−1^ with R^2^ value of 0.998. The higher rate is mostly due to the higher calorimetric yield of the device used, especially since the rate constant of the PFOS degradation is lower in their case (about 0.0153 min^−1^). Experiments under 400 W/L conditions were not conducted for all of the parameters due to the limitation in reactor configuration. Considering identical conditions (200 W/L power density and 580 kHz frequency), our reaction rate was found to be higher and E_EO_ was lower (2340 vs 700 kWh/m^3^), which could be attributed to intense cavitation generation via unique reactor design and proper optimization of reaction parameters via the integrated sonochemical system.

### 3.2 Degradation and mineralization extent

**Fig. 4:**
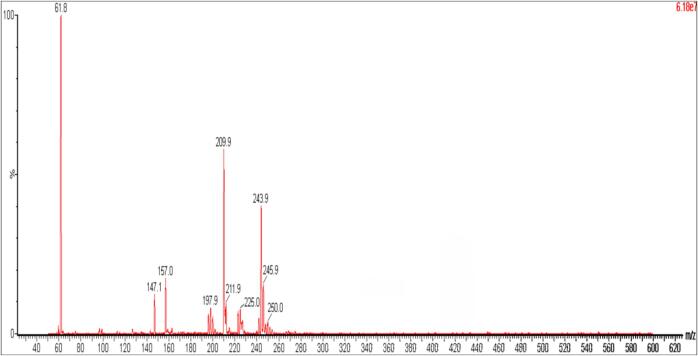
Mass spectra of intermediate products formed after 60 min sonication.

The PFOS removal progress was monitored via UPLC-MS/MS. Based on the PFOS disappearance outcomes, attempts have been made to characterize the degradation by-products by performing the full-scan mass spectrometry analysis (Fig. 4). Even if low-resolution mass spectrometry analysis doesn’t allow the identification of by-products, some fragments have been identified as: (a) 62 *m*/*z*, (b) 147 *m*/*z*, (c) 157 *m*/*z*, (d) 210 *m*/*z* and (e) 244 *m*/*z*, which belong to the by-products. The fragments were identified in the dead volume of the chromatogram, which indicated that there was no retention on the chromatographic column and the fragments eluted simultaneously. Only linear structures that could arise during the PFOS bond scission have been proposed while other related PFAS short-chain structures have been disregarded. These compounds are therefore polar and lost the amphiphilic nature of PFOS followed by rapid scission via sonochemical treatment. The appearance of degraded products subsequently disappeared with a longer duration of treatment until 180 min. The intermediate products are believed to undergo a similar degradation mechanism as PFOS until the complete mineralization.

**Fig. 5:**
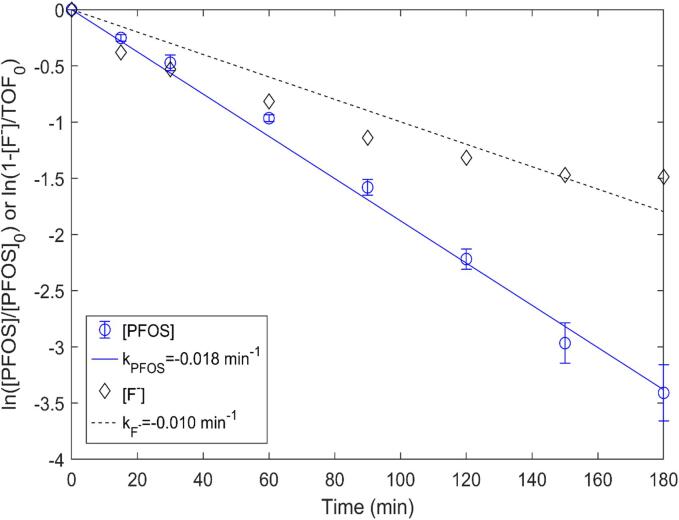
Comparative outcome of PFOS removal vs mineralization.

**Fig. 6:**
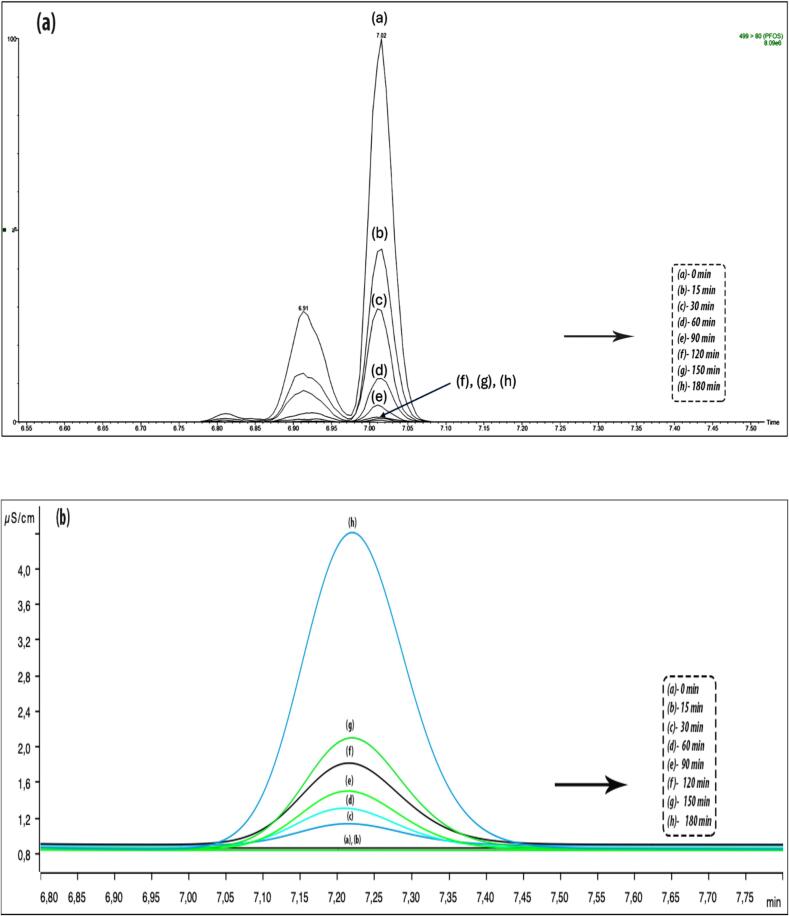
Comparative outcome of PFOS (a) concentration decline vs (b) fluoride evolution.

The extent of removal determined by UPLC-MS/MS analysis and mineralization (fluoride ion release) measured by IC analysis were very close to each other, indicating complete removal of PFOS using sonochemical treatment. Under optimum conditions, the degradation percentage determined by UPLC-MS/MS was 100 % and that for the IC was 99 %. The comparative outcome is depicted in Fig. 5. The concentration of fluoride (F^-^) started to increase with sonication and reached maximum by the end of treatment. Fig. 6 shows the decline in PFOS concentration over the treatment time obtained from mass spectrometry and the fluoride evolution obtained from IC analysis. The maximum percentage of PFOS removal at 400 W/L was found to be 100 % for the initial concentration of 5 mg/L, while the corresponding percentage of defluorination was 98 %. Similarly, the maximum percentage of PFOS removed at 267 W/L at an initial concentration of 3 mg/L was 99 %, and the corresponding percentage of defluorination was 98 %. The maximum percentage of PFOS removal was 97 % for 1 mg/L at 267 W/L with a corresponding defluorination of 95 %. Considering 5 mg/L PFOS under 267 W/L, the removal was 97 % and defluorination was 88 %. For each of the three initial concentrations, the difference between the percent PFOS removal and the percent defluorination was not significantly different.

Although certain existing studies have also reported working on the sonochemical removal of PFOS, it is imperative to provide a comprehensive overview of their work and the shortcomings, for which the current work holds significance. Therefore, Table 2 lists relevant work on PFOS with our added comment, which shows how this work could serve as a benchmark study for the future while looking into the inconsistencies in earlier works. Even though the listed studies did not include all precise information, looking into the report by Wood et al. [14] (k: 0.013 min^−1^) and Kewalramani et al. [16] (k: 0.004 min^−1^), where identical conditions (concentration, liquid volume, frequency) were somehow adopted, our work demonstrated higher efficiency (k: 0.02 min^−1^) in PFOS removal. However, it is inappropriate to consider the listed data in the Table 2 for comparison because the slightest change in power could affect the PFOS removal output.

**Table 2:** PFOS sonolytic removal under high-frequency operations.

Ultrasound frequency (*f*) (kHz)	Other parameters	Comment	Reference
500	C_0_: 2 µg/L–5 mg/L P (W/L): 200–400 Sol. Temp.: 25–35 °C Cal. Yield (%): 31 k(min^−1^): 0.010–0.031 Removal (%): 95–100 Defluorination (%): 99 Duration (h): 3	(1) Precise calorimetric measurement along with yield are presented. (2) Both removal and defluorination extent are presented.	This work
700 + 900	C_0_: 0.5–10 mg/L P (W/L): 90–145 Removal (%): 31–47 Defluorination (%): 28–51 Duration (h): 6	(1) No information on rate constant. (2) No calorimetric measurements.	[12]
500	C_0_: 5–230 mg/L Sol. Temp.: 30 °C	(1) No information on rate constant. (2) No calorimetric measurements.	[13]
500	C_0_: 10 mg/L Volume: 200 mL Sol. Temp.: <45 °C k (min^−1^): 0.013 Removal (%): 93.8 Duration (h): 4	(1) No calorimetric yield/efficiency information.	[14]
575	C_0_: 50 µg/L P (W/L): 262 Sol. Temp.: 30 °C k (min^−1^): 0.065 Duration (h): 2	(1) No calorimetric yield/efficiency information.	[15]
700	C_0_: 5.7 mg/L P (W/L): 100 Sol. Temp.: <45 °C k (min^−1^): 0.004 Duration (h): 4	(1) No calorimetric yield/efficiency information. (2) No defluorination measurement.	[16]
354	C_0_: 100 ug/L P (W/L): 250 k (min^−1^): 0.027 Duration (h): 2	(1) No calorimetric yield/efficiency information.	[17]
200	C_0_: 10 mg/L Volume: 60 mL Sol. Temp.: 20 °C k (min^−1^): 0.0068 Duration (h): 1	(1) No calorimetric measurements.	[18]
612	C_0_: 100 ug/L P (W/L): 250 Sol. Temp.: 10 °C k (min^−1^): 0.0135 Duration (h): 2	(1) No calorimetric yield/efficiency information.	[19]

All experiments were conducted under simulated water environment; C_0_ : Initial PFOS concentration; P: Power density; Sol. Temp.: Solution Temperature; Cal.: Calorimetry; k: Rate constant.

Considering the mineralization outcome and the appearance of degraded products starting from higher to lower molecular weight, the successive dissociation of CF_2_ and the loss of sulfonate functional group from PFOS followed by the formation of fluorinated intermediates until complete defluorination is assumed to take place during sonication. During the mineralization, PFOS is expected to produce C1 fluoro-radicals, carbon monoxide, carbon dioxide and fluoride ions as final products. PFOS is known as an anionic surfactant due to its hydrophilic (acid) tail and hydrophobic (perfluoroalkyl) head. The sonochemical degradation of a pollutant could take place in three different phases. The pollutant could travel to the bubble core and get pyrolyzed. It could also be present in bubble–liquid interfacial regions to follow thermal decomposition or remain in the liquid bulk phase for radical-driven degradation. The degradation mechanism is dependent on the nature of the molecule. As PFOS is nonvolatile, the chance of being transported into the bubble core and undergoing pyrolysis is limited. Therefore, the first dominant degradation pathway could be attributed to the interfacial thermal decomposition of PFOS followed by pyrolysis of short-chain degraded products inside the bubble-core, resulting in complete mineralization [21]. Considering the faster mineralization rate of PFOS, the role of free radicals should be considered as negligible. For pollutants such as PFOS, which has very stable chemical bonds, high-temperature pyrolysis plays a vital role in its mineralization. The oxidizing radicals could not break down the stable C-F bond and initiate defluorination. Apart from that, the oxidative degradation pathway could result in a large number of intermediate formations, which could be adsorbed at the gas–liquid interface and hinder the pyrolytic degradation from taking place at the bubble core [17].

### 3.3 Effect of frequency on PFOS removal

The extent of PFOS removal could be significantly impacted by the operating ultrasound frequency. To understand the difference, experiments were conducted under 22 and 500 kHz frequencies while keeping constant all other optimum conditions. The outcomes indicated that operation under 22 kHz resulted in negligible and inconsistent PFOS removal as compared to 500 kHz operation. Under high-frequency operation, the bubbles could reach the resonance size faster, which ultimately enhances cavitation generation, resulting in a higher number of imploding bubbles and the corresponding increase in the number of reactive oxygen species available for pollutants to degrade faster. This effect is evident from the KI dosimetry (Weissler dosimetry) outcome included in this work, where the generation of OH• radicals under 500 kHz operation doubled compared to 22 kHz frequency. Shende et al. [24] reported the identical phenomena during the degradation of PFOS under high-frequency operation. They indicated that cavitation events increased under high-frequency operation and the PFOS degradation could be attributed to the thermal decomposition at the gas–liquid interface or inside the bubble core. Rodriguez-Freire et al. [13] also reported a similar PFOS removal trend, where a very slow degradation rate was witnessed under low frequency (25 kHz) compared to the high frequency (500 kHz, 1 MHz) operation. For a 10 µM PFOS solution, only 6.8 % of defluorination was obtained for 25 kHz operation after 180 min of treatment. However, under 500 kHz and 1 MHz operation, 11.4 times and 14.2 times higher defluorination was obtained, respectively, compared to the 25 kHz ultrasonication. It is vital to note that the defluorination efficiency under both 500 kHz and 1 MHz became identical for the PFOS concentration of 460 µM. It indicates that the frequency effect is also PFOS initial concentration dependent. Yet again, outcomes reported by Wood et al. [14] indicated that the PFOS degradation followed a descending order of 400 > 500 > 1000 kHz with 96.9, 93.8 and 91.2 % degradation and negligible degradation under 44 kHz. The decline in removal rate under high-frequency operation could be attributed to the concentration and saturation relationship. However, all high-frequency operations performed way better than the low-frequency for the investigated concentration range (6–10 mg/L). Considering the significant PFOS removal efficiency under high-frequency sonication compared to the low-frequency operation in our case, it could also be justified based on established facts on other pollutants. Meng et al. [25] performed sonochemical treatment of bisphenol A and reported higher radical generation and more efficient degradation of the pollutant under 400 kHz frequency compared with the 200 kHz, due to the more frequent cavitation generation and collapse event.

### 3.4 Effect of power density on PFOS removal

**Fig. 7:**
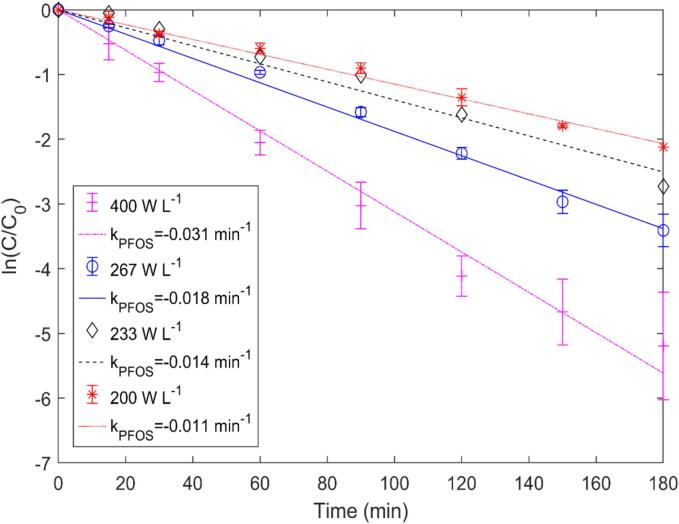
Time dependent removal efficiency under different power densities.

To understand the effect of ultrasonic power density on PFOS removal, experiments at different power densities (200–400 W/L) were conducted while keeping the solution temperature at 25 °C and initial concentration at 5 mg/L. The time-dependent removal efficiency based on different power densities is plotted in Fig. 7. As shown in Fig. 7, the sonochemical removal efficiency enhanced with the increase in power density for 500 kHz operation. The removal rate followed first-order reaction kinetics while demonstrating a rate constant of 0.011 min^−1^, 0.014 min^−1^, 0.018 min^−1^ and 0.031 min^−1^ for power densities of 200 W/L, 233 W/L, 267 W/L and 400 W/L, respectively, with R^2^ value consistently above 0.998. The rate of fluoride release also followed a similar trend, i.e., the defluorination rate increased with the increase in power density. Similar phenomena have been reported by Kewalramani et al. [16] and Marsh et al. [12], where the increase in ultrasonic power density resulted in a significant increase in PFOS removal. A faster PFAS removal rate was observed at a higher power density (145 W/L) compared to the lower power density (90 W/L) with an identical fluoride release trend. Similarly, Shende et al. [15] also reported a linear increase in PFOS degradation efficiency from 34 % to 97 % with the increase in power density (30–262 W/L).

The growth and collapse of bubbles as well as bubble–bubble interactions is also dependent on the ultrasonic amplitude. The bubble–bubble interactions increase with the rise in ultrasound amplitude, leading to highly responsive cavitation bubble oscillations to the secondary Bjerknes force [26]. The ultrasonic power, ultrasonic amplitude and sound intensity are all inter-connected and can be linked to each other in understanding the effect of power density on pollutant removal efficiency. As a fundamental aspect, the higher the ultrasonic power, the greater will be the localized pressure generated by ultrasound waves and the ultrasonic amplitude. The bubble undergoes cavitation only when the ultrasonic amplitude is higher than the threshold value [27]. The cavitation bubble size, bubble collapse time, transient temperature and internal pressure during the bubble collapse are all dependent on the power intensity. The power intensity (I) could be determined by the acoustic amplitude (P_A_), in the following way (Eq. 6):

(6) $$I=\frac{P_{A}^{2}}{2\rho c}$$

Where I (W/m^2^) is the sound intensity (amount of energy flowing per unit area per unit time), ρ is the density of the medium and c is the velocity of sound in the medium. With the increase in ultrasonic power density, the input ultrasonic energy in the reaction system as well as the bubble expansion ratio (R_max_/R_0_) increases. The bubble radius can expand to >20 times the equilibrium radius if the amplitude is sufficiently high. This results in the increase in active cavitation bubbles, the transformation of bubbles to the transient cavitation phase from stable cavitation, enhancement in potential energy to be converted into chemical reactions and heat during the bubble collapse event [28], [29]. Pandit et al. [30] reported an increase in the amount of ⋅OH radicals with the increase in ultrasound amplitude, which justifies the enhanced cavitation activity. However, very high power densities could produce a bubble shielding effect, which can negatively impact reactor performance.

### 3.5 Effect of initial concentration on PFOS removal

**Fig. 8:**
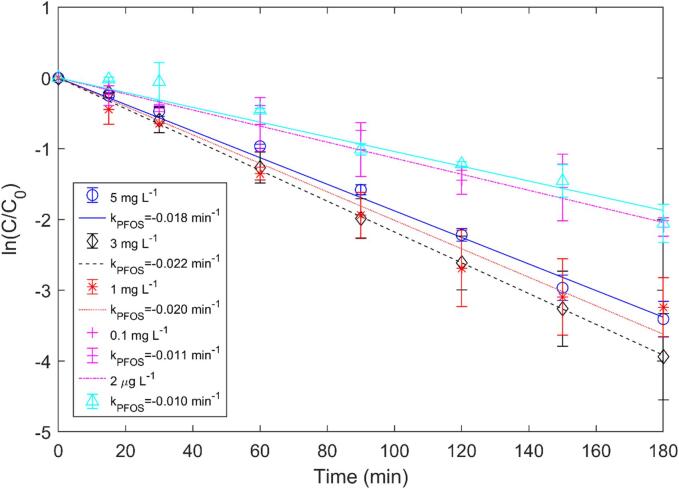
Effect of initial concentration on PFOS removal.

The PFOS removal efficiency could be affected by a change in its initial concentration. Several studies have shown how crucial the optimum initial PFAS concentration could be for the most effective removal [17], [31]. To understand the concentration effect on PFOS removal, it’s initial concentration was varied from 2 μg/L to 5 mg/L while maintaining solution temperature at 25 °C and power density at 267 W/L. Even though the highest removal rate (0.022 min^−1^) was obtained for 3 mg/L, the rate declined for 100 µg/L (0.011 min^−1^) and 2 µg/L (0.010 min^−1^) (Fig. 8). Therefore, it can be inferred that beyond an optimum value, the removal rate could alter.

The degree of transport, aggregation and pyrolytic destruction of semi-volatile or non-volatile contaminants at the bubble–liquid interface depends on their concentration [32]. Vecitis et al. [17] reported that saturation of adsorptive sites at the bubble interfacial region caused a decrease in PFOS degradation efficiency once the concentration exceeded 20 μM. Similarly, Marsh et al. [12] reported witnessing an increase in fluoride release with the increase in PFAS concentration up to 55 μM, beyond which there was no enhancement. Based on Rodriguez-Freire et al. [13], the defluorination rate also increased with the increase in PFOS concentration up to 100 μM, beyond which no enhancement occurred. These phenomena were attributed to the excessive foaming of PFAS at higher concentrations and the saturation of cavitation bubbles beyond the limit. However, there is no report on the effect of low concentration below the optimum value. Therefore, we have chosen low to high PFOS initial concentration to demonstrate the effect, which could guide future research in the right direction. Even though, we have also witnessed a similar removal trend with the increase in PFOS concentration beyond 3 mg/L, the decline in removal rate at both of the low concentrations could be attributed to the low availability of PFOS molecules for the bubble interface to undergo mineralization. Considering the high concentration range, it can be assumed that the bubble’s surface becomes saturated beyond the optimum concentration [32], resulting in a decline in the removal rate.

### 3.6 Effect of reaction temperature and pH

**Fig. 9:**
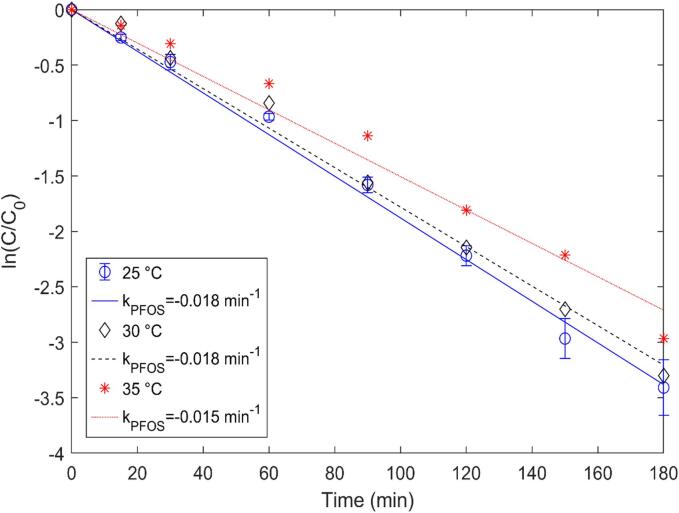
Effect of solution temperature on PFOS removal.

The cavitation intensity can be impacted by the change in reaction solution temperature and thereby the alteration in the physiochemical characteristics of the liquid medium [33]. Throughout this experiment, a chiller was used to regulate the reaction solution’s temperature between 25 and 35 °C and other essential parameters such as the PFOS initial concentration and ultrasonic power density were maintained to understand the role of temperature. The highest removal rate (0.018 min^−1^) was achieved at an operating temperature of 25 and 30 °C compared to 35 °C (0.015 min^−1^), which indicated that the rise in temperature above the optimal value marginally reduced the PFOS mineralization rate (Fig. 9). The cavitational intensity and type of cavities (vaporous or gaseous) being formed are dependent on the solution temperature [34]. At high solution temperatures, the bulk phase PFOS could be easily transported to the bubble interface, but the mineralization efficiency may suffer from the subsequent decrease in collapse intensity with the rise in temperature beyond the optimum value. A similar outcome was reported by Lin et al. [35] and Yang et al. [36], where the PFOS degradation reduced at the elevated solution temperature.

**Fig. 10:**
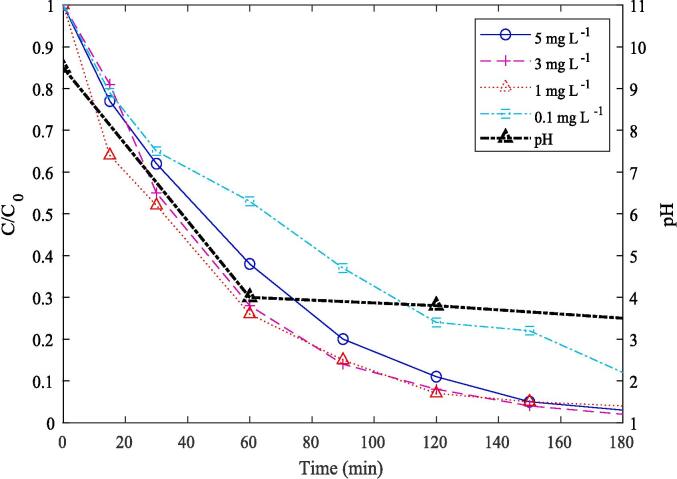
Relation between solution pH and PFOS removal.

The solution pH was not maintained at any specific value regardless of earlier reports suggesting to conduct experiments under an acidic environment. This procedure was adopted to mimic the real-world treatment scenario where operating at a specific pH won’t be feasible. The pH of the reaction solution was measured continuously until the end of the experiment, which was found to decline from 9.5 to 3.5 consistently for each experiment. The pH declined to 4 within just 60 min of treatment, followed by a slight decrease until 180 min (Fig. 10). It shows that PFOS was effectively converted from its initial salt form (K-PFOS) to the acidic form within just 60 min of treatment. Considering the degradation trend of higher initial concentration (1, 3 and 5 mg/L), it could be observed that the PFOS concentration declined up to 70 % (average) within 60 min of treatment under optimal conditions. This could be attributed to the PFOS in its initial salt form, which is more soluble in water and the increased solubility could make the compound more susceptible to degradation under cavitation, as it can more readily interact with radicals and high-energy environments. The decline in degradation rate after 60 min of treatment and correlating it to the measured pH value, illustrates that under acidic conditions, cavitation may be less effective due to the limited solubility of the PFOS in its acidic form. Considering the low initial concentration (2 and 100 µg/L), the PFOS concentration declined to 40 % (average) within the 60 min of treatment and further declined equivalently as of high initial concentration, demonstrating the impact of PFOS in its acidic form after 60 min of treatment. This corresponds to the degradation as well as mineralization trend obtained via UPLC-MS/MS and IC analysis, where the removal rate reached 90 % within 120 min of treatment. The decline in pH could be attributed to the likely formation of radicals and numerous acid species such as HF, carbonic acid from dissolved CO_2_ as well as nitrous and nitric acids from air-saturated systems [37]. A similar outcome was also reported by Wood et al. [14], where the pH declined from the initial value of 5.66 to 3.16 within 4 h of PFOS (salt form) treatment via 500 kHz frequency. Ilić et al. [20] also indicated a decline in the pH value of the PFOS (acid form) reaction solution from 7 to 3.6 during the 580 kHz treatment. Considering the report by Kewalramani et al. [16], the PFOS (salt form) and PFOA solution’s initial pH (5 to 5.5) dropped to a range of 3.8–4.5, resulting in a more acidic solution at the end of the 4-hour treatment. Even though earlier reports demonstrated the decline in pH over treatment time irrespective of the nature of PFOS starting material (acid or salt), our work illustrated the progressive removal efficiency of PFOS over treatment time and the relationship between the solution pH and cavitation.

## 4 Conclusions

As most of the sonochemical-based PFAS treatments were conducted in laboratory environments using inaccurate parameter measurements, this study focused on accurately determining the removal efficiency. The sonochemical treatment of PFOS was performed under both low (22 kHz) and high-frequency (500 kHz) while maintaining other optimum conditions. Vital conclusions can be drawn as follows:

• High-frequency (500 kHz) operation resulted in the complete removal of PFOS within just 180 min of treatment. Low-frequency (22 kHz) operation resulted in inconsistent and insignificant removal under identical operational conditions.
• To compare the removal extent with earlier works and provide a benchmark study for future studies, calorimetric measurements and electrical energy per order (E_EO_) were determined. The outcome indicated higher removal efficiency (k: 0.031 min^−1^) compared to most of the earlier works that reported the removal rate constant or energy efficiency calculation.
• The PFOS removal efficiency was monitored via state-of-art UPLC-MS/MS analysis and the degraded products were proposed based on the full-scan analysis. The defluorination extent was measured using IC analysis and was found to match the removal rate.

Future research could consider this work while deciding the upscaling potential of batch-type sonochemical treatment as it provides all the required information. In addition to energy consumption and cost, a number of other parameters need to be taken into consideration. Therefore, careful optimization of the bench-scale studies is required which might be dependent on the ultrasound reactor geometry and size, the transducer’s size, and the physicochemical characteristics of the aqueous matrix. It is critical to realize that the limited zones of efficacy for cavitation bubble propagation from transducers may present considerable design issues for scale-up. Thus, more research and development are required to bring this technology to a field-trial state.

## 5 Statement of novelty

The sonochemical removal of PFOS in a simulated aqueous solution has been critically investigated in this study while considering the energy aspects and all other parameters necessary for the upscaling potential. This work has shown how the precise measurement of power and energy calculation is vital for deciding the removal efficiency, which has been missing in most of the earlier works. Overall, this work has shown better PFOS removal efficiency compared to comparable works, which could be attributed to the unique reactor configuration and the ability to control the reaction with an advanced integrated software system. As a result, this study can be considered as a benchmark reference for any PFAS or PFOS-related future work.

## CRediT authorship contribution statement

**Debabrata Panda:** Writing – review & editing, Writing – original draft, Visualization, Validation, Methodology, Investigation. **Maxime Cochennec:** Writing – review & editing, Supervision, Methodology, Data curation. **Stéfan Colombano:** Writing – review & editing, Supervision, Project administration, Funding acquisition. **Benjamin Laulier:** Visualization, Validation, Software, Resources, Conceptualization. **Pascal Tierce:** Software, Resources. **Alexandre Baudouard:** Software, Resources. **Sebastian Bristeau:** Formal analysis, Data curation. **Anne Togola:** Writing – review & editing, Formal analysis, Data curation. **Julie Lions:** Writing – review & editing, Supervision, Funding acquisition. **Nicolas Devau:** Project administration. **Eric D. van Hullebusch:** Writing – review & editing, Supervision, Project administration, Methodology, Investigation, Funding acquisition.

## Declaration of competing interest

The authors declare that they have no known competing financial interests or personal relationships that could have appeared to influence the work reported in this paper.
